# Decreasing Production and Potential Urban Explosion of Nighttime Nitrate Radicals amid Emission Reduction Efforts

**DOI:** 10.1021/acs.est.3c09259

**Published:** 2023-12-08

**Authors:** Yuhang Wang, Shengjun Xi, Fanghe Zhao, Lewis Gregory Huey, Tong Zhu

**Affiliations:** † School of Earth and Atmospheric Sciences, Georgia Institute of Technology, Atlanta, Georgia 30332, United States; ‡ State Key Joint Laboratory of Environmental Simulation and Pollution Control, College of Environmental Sciences and Engineering, Peking University, Beijing 100871, China

**Keywords:** nighttime oxidation, nocturnal nitrate radical production, odd oxygen, nitrogen oxides, ozone

## Abstract

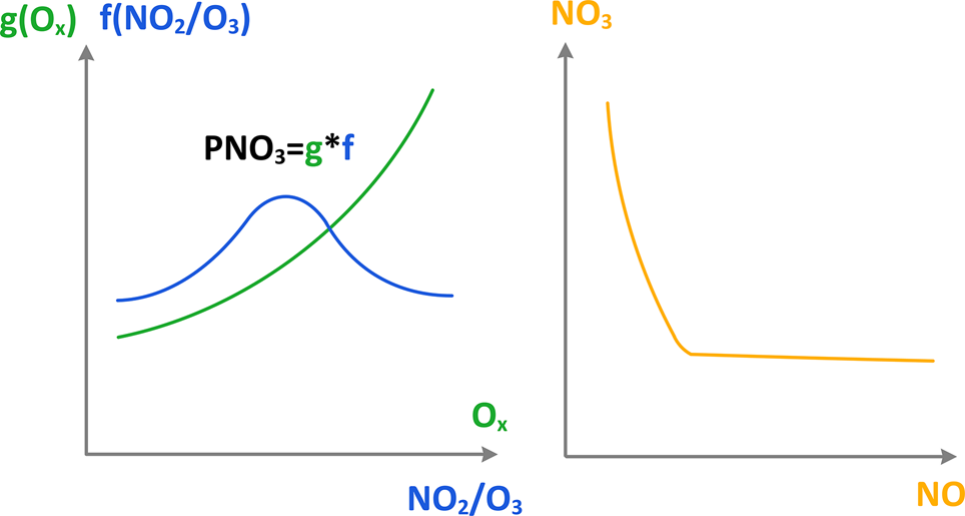

Nighttime oxidation by nitrate (NO_3_) radicals has important ramifications on nocturnal aerosol formation and hence the climate and human health. Nitrate radicals are produced by the reaction of NO_2_ and O_3_. Despite large decreases in anthropogenic emissions of nitrogen oxides (NO_*x*_ = NO + NO_2_), a previous study found significant increases in NO_3_ production (PNO_3_) from 2014 to 2019 in China, in contrast to decreasing trends in the U.S. and Europe. Using the summer observations from 2014 to 2022, we analyze the interannual variability of nocturnal PNO_3_ using a systematic framework, in which PNO_3_ is diagnosed as a function of odd oxygen (O_*x*_ = O_3_ + NO_2_) and the NO_2_/O_3_ ratio. We did not find an increase of PNO_3_ from 2014 to 2022 in China due to a continuous decrease in the NO_2_/O_3_ ratio, although PNO_3_ is modulated by the variation in O_*x*_. Using in situ observations obtained in Beijing in 2007, we demonstrate the potential for an upsurge resembling an “explosion” in urban nighttime NO_3_ radicals amid emission reduction efforts.

## Abstract 2

Emission reduction efforts will result in a decrease in nighttime PNO_3_ and have the potential to cause upsurges in nighttime nitrate radicals for certain urban areas.

## 1 Introduction

Nocturnal oxidation by nitrate (NO_3_) radicals can produce significant amounts of secondary aerosols affecting climate and human health.^1−4^ While in situ measurements of NO_3_ have been made for decades,^5^ long-term records of nighttime NO_3_ are not available due in part to the short lifetime and high variability of its concentrations,^6^ making it difficult to assess the changes of nocturnal oxidation in the past due to anthropogenic emission changes in response to environmental regulations. However, the production rate of NO_3_, PNO_3_, near the surface can be diagnosed using observations of NO_2_ and O_3_ by air quality monitoring networks around the world since the reaction R1 is the main source of NO_3_ in a polluted boundary layer.^1^
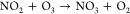
R1

In a recent study, Wang et al. analyzed the trend of nocturnal PNO_3_ in the warm season of China from 2014 to 2019 in comparison to those in the United States and Europe.^1^ They found a significant increasing trend of nocturnal PNO_3_ in China in contrast to the decreasing trends in the United States and Europe and further suggested that nocturnal oxidation is becoming more important in China. In this study, we analyze the variation of nighttime PNO_3_ in China from 2014 to 2022 using a systematic framework to separate the effects of odd oxygen (O_*x*_ = O_3_ + NO_2_) and the ratio of NO_2_/O_3_.

Given the short lifetime of NO_3_, PNO_3_ can often be used to assess the nighttime NO_3_ radical oxidation potential in summer.^1^ However, the NO_3_ concentration and its oxidation rate of volatile organic compounds (VOCs) also depend on two other losses. The reaction of NO_2_ and NO_3_ produces N_2_O_5_, which thermolyzes back into NO_2_ and NO_3_ with a lifetime of ∼1 min at 20 °C.^7^ Heterogenous reactions of N_2_O_5_ that produce HNO_3_ and ClNO_2_ on aerosols remove NO_3_ radicals from the atmosphere.^2^ In addition, NO_3_ is also removed by reaction with NO. We examine the potential impact of urban nighttime NO on NO_3_ concentrations using in situ observations.

## 2 Materials and Methods

**Figure 1:**
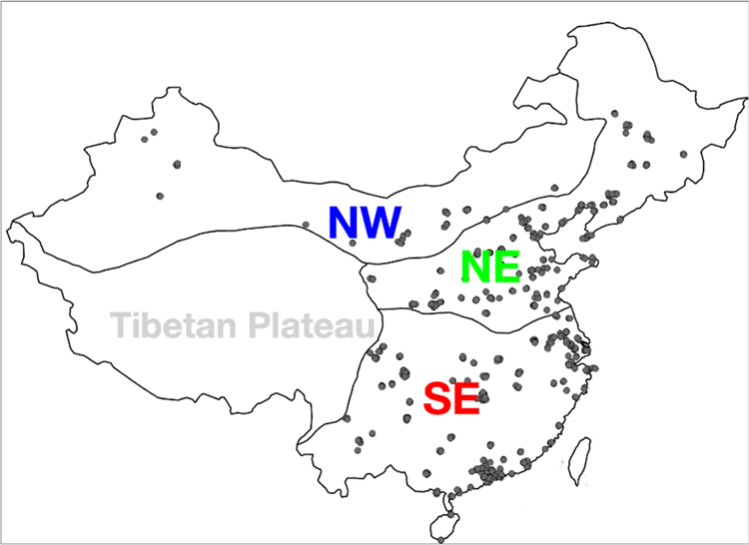
CNEMC sites (black dots) with continuous measurements from 2014 to 2022 in three analysis regions.

Similar to the previous study,^1^ we use the hourly observations of surface NO_2_ and O_3_ between 8 pm and 5 a.m. LT (at altitudes typically a few meters above the surface) by the China National Environmental Monitoring Center (CNEMC) network. To ensure data consistency, we only analyze summertime (June, July, and August) data from 2014 to 2022. Limiting the analysis to summer data ensures a more consistent physical and chemical environment. The ending time of nighttime analysis is changed from 6 am^1^ to 5 am for the summer. We use only the data from stations that were continuously operational from 2014 to 2022. The observations from 640 surface stations are used in the analysis. Furthermore, we filter out the outlier data outside the range of *Q*_1_ – *k(Q*_3_ – *Q*_1_) and *Q*_1_ + *k(Q*_3_ – *Q*_1_), where *Q*_1_ and *Q*_3_ are the 25th and 75th quartiles, respectively, and *k* = 1.5.^8^ 2.65% of the measurement data are removed, although this filtering does not alter the analysis results. Lastly, to further ensure data consistency, we group the observations in China into 4 regions based on climate characteristics and topography (Figure 1). There are only 6 stations over the Tibetan Plateau region, and hence only data in the Northeast (NE), Southeast (SE), and Northwest (NW) regions are analyzed.

Chemical reaction rate constants depend on the atmospheric temperature and pressure. We use the European Centre for Medium-Range Weather Forecasts (ECMWF) Reanalysis version 5 (ERA5) hourly surface temperature and pressure at a resolution of 0.25° × 0.25°. Reaction rate constants are calculated using the kinetics data from the latest Jet Propulsion Laboratory (JPL) compilation.^7^

To add a point of reference for the pre-2014 period, we analyzed the observations from the Campaigns of Air quality Research in Beijing (CAREBeijing) in August 2007. Concentrations of O_3_, NO, and NO_2_ were measured at an urban site located on a building rooftop (∼20 m above the ground level) on the campus of Peking University.^9,10^ The 1 minute observations were averaged hourly to be comparable to the CNEMC measurements.

## 3 Results and Discussion

### 3.1 Systematic Analysis Framework

In polluted regions at night, O_3_ is consumed to oxidize freshly emitted NO:
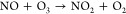
R2

**Figure 2:**
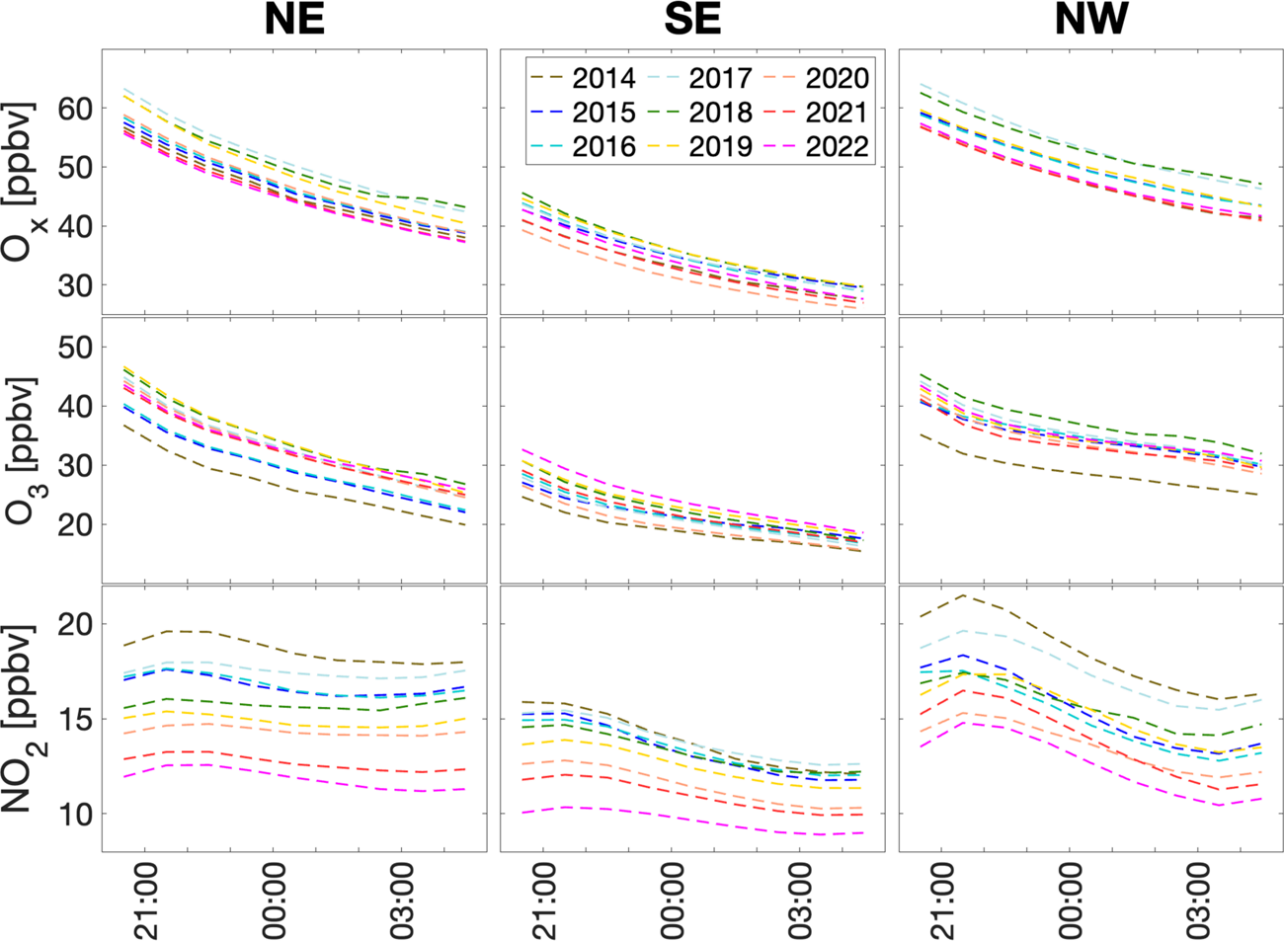
Summertime hourly averaged regional O_*x*_, O_3_, and NO_2_ concentrations at 8 pm to 5 am LT from 2014 to 2022 in the NE, SE, and NW regions. The hourly data points are plotted in the middle of the hour. The corresponding standard deviation distributions among the observation sites are shown in Figure S1.

We define nighttime odd oxygen as O_*x*_ = O_3_ + NO_2_.^11^Figure 2 shows the summertime hourly averaged regional O_*x*_, O_3_, and NO_2_ concentrations from 2014 to 2022. O_*x*_ concentrations decreased at night largely following the decreases of O_3_ since the variation of NO_2_ was relatively small compared to those of O_*x*_ and O_3_. The nighttime O_3_ and NO_2_ variation patterns in China are similar to those observed in the United States.^12^

We write the PNO_3_ rate as a function of O_*x*_,
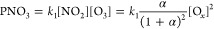
1where  and *k*_1_ is the reaction rate constant of R1. In this analysis framework, PNO_3_ is proportional to [O_*x*_]^2^ and *f*(α), where . The maximum of *f*(α) is 1/4 when α = 1.

### 3.2 Summertime Variations of PNO_3_

**Figure 3:**
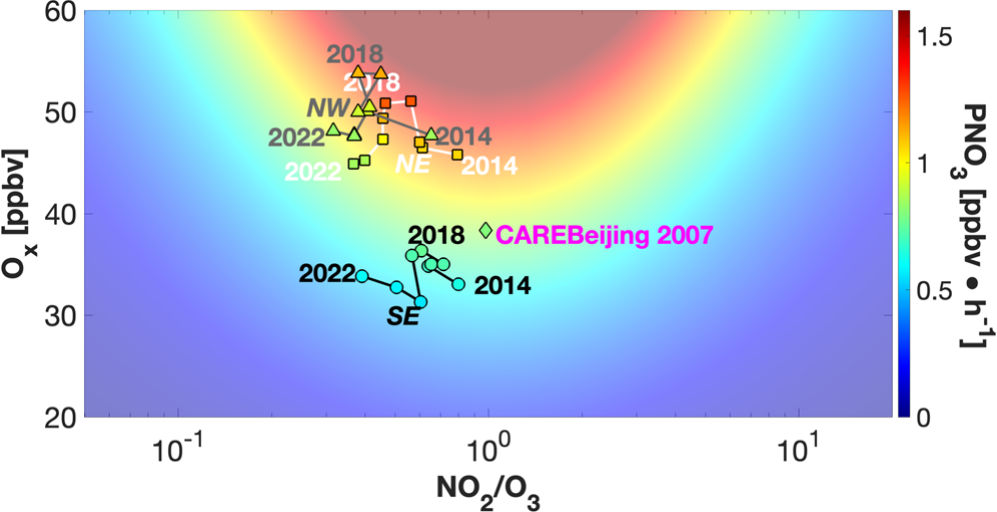
Regional averaged summer PNO_3_ as functions of the geometric mean of NO_2_/O_3_ and average O_*x*_ from 2014 to 2022 in the NE (squares), SE (circles), and NW (triangles) regions. The background color image was computed using eq 1 at a temperature of 20 °C. PNO_3_ was computed using hourly CNEMC measurements and ERA5 temperature. Therefore, regional nighttime average PNO_3_ values do not necessarily match those in the background color image. Averaged PNO_3_ for the CAREBEIJING-2007 campaign is denoted by a diamond. The geometric mean of NO_2_/O_3_ is used since it is in log scale.

Diagnosing PNO_3_ as a function of O_*x*_ and NO_2_/O_3_ ratio makes it easier to understand PNO_3_ variations from 2014 to 2022 than the method used in the previous study.^1^ The averages of summertime PNO_3_ from 2014 to 2022 are plotted as a function of NO_2_/O_3_ and O_*x*_ in Figure 3. The background color image in the figure was computed using eq 1, which shows that PNO_3_ has a quadratic dependence on O_*x*_. However, the dependence of PNO_3_ on the NO_2_/O_3_ ratio is more complex: it decreases with decreasing NO_2_/O_3_ ratio when the ratio is <1, but increases with decreasing NO_2_/O_3_ ratio when the ratio is >1. In the regions of this study, the NO_2_/O_3_ ratio was <1 and generally decreased with time. Consequently, PNO_3_ in all regions primarily decreased from 2014 to 2022 due mostly to the decrease in the NO_2_/O_3_ ratio. PNO_3_ values in the three regions had similar variations (Figures 3 and S2). Between 2014 and 2022, the peaks in summertime PNO_3_ rates occurred around 2017–2018 due to the greater effect of the increase in the level of O_*x*_ since 2014 compared to the decrease in the NO_2_/O_3_ ratio. After peaking around 2017–2018, O_*x*_ concentrations decreased to and even went below the 2014 O_*x*_ level during the 2020–2022 period, while the decrease in the NO_2_/O_3_ ratio continued. As a result, PNO_3_ rates in all three regions decreased to levels below those observed in 2014. The modulation effect by O_*x*_ is particularly large from 2020 to 2022 in the SE as the increase of O_*x*_ in these 3 years is faster than during any 3-year periods of 2014–2019 (Figure S2). However, the resulting increase in PNO_3_ is slight (Figure S2) due to the decrease of the NO_2_/O_3_ ratio from 2020 to 2022 (Figure 3).

**Figure 4:**
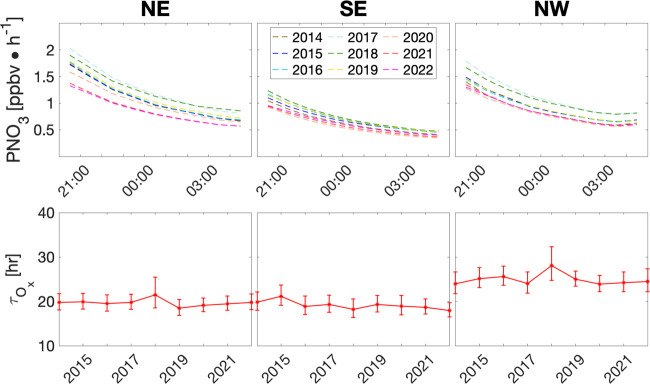
Same as Figure 2 but for hourly average PNO_3_ rate and the corresponding O_*x*_ lifetime. The corresponding standard deviations of PNO_3_ among the observation sites are shown in Figure S4.

Figure 4 shows that hourly average PNO_3_ rates for the three regions decreased from 2014 to 2022 at night. The variation of PNO_3_ at night is larger than in daytime in summer.^13^ The intranight decrease of PNO_3_ by a factor of ∼2 was larger than those of O*_x_*. It was driven primarily by the decay of O_*x*_ due to the quadratic dependence of PNO_3_ on the O_*x*_ concentrations (eq 1). Quantifying nighttime O_*x*_ decreases is therefore necessary.

Figure 2 shows the nearly exponential decay of the O_*x*_ concentrations at night in all three regions. The decay shapes were fairly consistent among different years, i.e., the seasonal mean difference at 8 pm LT was largely maintained until 5 am LT. To quantify the nighttime decay lifetime, we conducted least-squares regressions using *x* = *x*_8pm_*e*^–*t*/τ^, where *x* is O_*x*_ hourly concentration, *t* is the time lapse from 8 pm, and τ is the decay lifetime. The regressions have R^2^ values in the range of 0.97–0.99. The lifetime of O_*x*_ is ∼20 h in the NE and SE regions, but is higher at ∼30 h in the NW region (Figure 4), likely reflecting higher canopy dry deposition of O_3_ and NO_2_ in eastern China than the NW. The vegetation coverage of eastern China is much higher than the NW,^14,15^ resulting in larger deposition of O_3_ and NO_2_.^16,17^ Monthly mean midnight dry deposition velocities for O_3_ and NO_2_, simulated using a regional chemical transport model,^12,18,19^ are much higher in the NE and SE than the NW region (Figure S3). Model description and further discussion on the loss of O_*x*_ due to dry deposition can be found in Supporting Information.

We apply least-squares regression analysis to compute interannual trends of O_*x*_ lifetimes. The SE region is the only region with a statistically significant small decreasing trend, −1.3% year^–1^ (*p* = 0.03) from 2014 to 2022. The other two regions also have negative trends, −0.28% year^–1^ (*p* = 0.63) for NE and −0.20% year^–1^ (*p* = 0.80) for NW. Moreover, taking into account significant seasonal variances that were larger than the trends, it is evident that nighttime O_*x*_ lifetimes remained largely unchanged despite substantial reductions in emissions across the three regions from 2014 to 2022, leading to fairly consistent nighttime decay patterns of O_*x*_ and PNO_3_ among different years (Figures 2 and 3).

The finding of stable nighttime lifetimes of the O_*x*_ from 2014 to 2022 is somewhat surprising considering the significant interannual variation of PNO_3_ (Figure 3). Since the lifetime of NO_3_ is short, PNO_3_ equals the loss rate of NO_3_, which includes (1) heterogeneous reactions of N_2_O_5_ on aerosols to produce HNO_3_ and ClNO_2_, (2) reactions of NO_3_ and VOCs, and (3) the reaction of NO_3_ and NO. In the first reaction pathway, up to three O_3_ molecules are lost for every N_2_O_5_ molecule that is lost. In the second reaction pathway, up to two molecules of O_3_ are lost for every NO_3_ loss. The third reaction pathway did not affect O_*x*_. Assuming the regional effect of the third reaction pathway was insignificant, an increase of PNO_3_ from 2014 to 2017–2018 (Figure 3) would imply a decrease of O_*x*_ lifetime, and the decrease in PNO_3_ from 2017 to 2018 to 2022 would have the opposite effect. However, the derived O_*x*_ lifetimes in the NE and NW regions had a peak in 2018 when PNO_3_ values were among the highest. The lack of correspondence between the O_*x*_ lifetime and PNO_3_ reflects in part the dominance of dry deposition of O_3_ and NO_2_ in determining the lifetime of the O_*x*_ at night.

Another possible contributor is that the rate of N_2_O_5_ heterogeneous reactions on aerosols decreased relative to the rate of the NO_3_ oxidation of VOCs. The loss of O_*x*_ through the latter is less than that through the former. The decrease in NO_2_ concentrations (by 31–37%) from 2014 to 2022 (Figure 2) reduced the formation rate of N_2_O_5_ considering that regional averaged PNO_3_ rates decreased. Furthermore, the heterogeneous loss rate of N_2_O_5_ depends on aerosol surface area. There was no regulatory monitoring of aerosol surface area. However, its qualitative trend can be estimated using PM2.5 concentrations by assuming that aerosol size distribution did not change significantly. Figure S5 shows that PM2.5 concentrations decreased by nearly a factor of 2 from 2014 to 2022 in the three regions. In addition, aerosol liquid water content also tends to decrease with PM2.5,^20^ contributing to a decrease of aerosol surface area. The overall effect was a decrease of the aerosol N_2_O_5_ reaction rate relative to the rate of NO_3_ oxidation of VOCs, which tended to decrease O_*x*_ loss and increase O_*x*_ lifetime at night.

Temperature at night decreases significantly and reduces the PNO_3_ rates. Figure S6 shows summertime hourly average temperatures from 2014 to 2022. Similar to that of O_*x*_, the interannual difference in average temperature is much less than the decrease in temperature from 8 pm to 5 am LT and the patterns of hourly temperature decrease are consistent among different years in the three regions. The resulting effect of interannual temperature variation on PNO_3_ rates is small from 2014 to 2022, although lower temperature in the NW resulted in lower reaction rate constants for PNO_3_ compared to those in the SE and NE regions (Figure S7).

### 3.3 Potential Explosion of Urban NO_3_ Radicals

Higher PNO_3_ rates in the NE and NW than the SE region are due largely to regional differences in O_*x*_ concentrations (Figure 3). The decrease in PNO_3_ rates from 2014 to 2022, on the other hand, is due mostly to the decreasing NO_2_/O_3_ ratio, reflecting much larger and more consistent NO_2_ reductions compared to the variations of O_*x*_ and O_3_ in the three regions (Figure 2). We did not find evidence that nighttime variations of chemical species or temperature, which were much larger than their interannual variations, changed significantly from 2014 to 2022. The lifetimes of nighttime O_*x*_ in the three regions did not show significant changes either (Figure 4).

While we find increases in PNO_3_ rates from 2014 to 2017–2018 in all three regions as Wang et al.^1^ due to increased O_*x*_ concentrations, the increases appeared to be transitory in nature due to persistent and rapid decreases in the NO_2_/O_3_ ratio as NO_*x*_ emissions were reduced. Some of the post-2020 NO_*x*_ emissions decreases are COVID-related.^21^ Nonetheless, it is possible that the rates of nighttime PNO_3_ increased prior to 2014. Sun et al. showed an increase of about 9.6 ppbv of maximum daily 8 h average O_3_ concentrations in July and August from 2003 to 2015.^22^ They attributed about half of the O_3_ increases to increased emissions of the NO_*x*_ and VOCs. Although the interannual variability of O_3_ between 2003 and 2015 was not studied, it is likely that nighttime PNO_3_ increased from 2003 to 2015 given the quadratic dependence of PNO_3_ on O_*x*_. Furthermore, Ding et al. showed increasing lower-tropospheric O_3_ concentrations in Beijing and the North China Plain region from 1995 to 2005.^23^

It is instructive to compare the 2014–2022 results for the NE region to the CAREBEIJING-2007 observations. The average PNO_3_ rate at night during the CAREBEIJING-2007 campaign was lower than the summertime average in the NE region (Figure 3) mostly due to a lower average O_*x*_ concentration. The geometric mean NO_2_/O_3_ ratio during the campaign was 1.3. Considering that the measurement altitude of 20 m is above air quality monitoring stations, it is possible that this measurement may underestimate near-surface NO/NO_2_ ratios due to the conversion of NO to NO_2_ during mixing from the surface to the measurement altitude. If this value was representative of the summer of 2008, the decrease in the NO_2_/O_3_ ratio from 2008 to 2014–2002 would slightly increase PNO_3_ until the NO_2_/O_3_ ratio reached 1. After this point, the decrease in the NO_2_/O_3_ ratio would start decreasing PNO_3_. However, this effect would not be as large as the increase in PNO_3_ due to increasing O_*x*_ (eq 1 and Figure 3).

Both historical observations of O_3_^22,23^ and CAREBEIJING-2007 measurements indicated that O_*x*_ concentrations increased prior to 2013 as anthropogenic emissions of NO_*x*_ and VOCs increased. The quadratic dependence of nighttime PNO_3_ on O_*x*_ implies that PNO_3_ increased during this period. However, it does not imply that nighttime NO_3_ concentrations increased, particularly in urban regions. CAREBEIJING-2007 1 minute measurements had 57% of nighttime NO concentrations >1 ppbv. At this level, the steady-state NO_3_ radical concentration is negligible due to the rapid removal of NO_3_ by NO,^7^
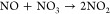
R3

NO concentrations were not reported by the CNEMC network. It is therefore difficult to quantify the effect of R3 in suppressing nighttime NO_3_ radical concentrations in urban areas with high NO_*x*_ emissions. Figure S8 shows that hourly average O_3_ concentrations during the CAREBEIJING-2007 campaign stayed at ∼10 ppbv after midnight and were lower than hourly average NO_2_ concentrations. The observed high NO_2_/O_3_ ratios during CAREBEIJING are due to high concentrations of NO, which reacts with O_3_ to produce NO_2_. We therefore use an indirect measure, the data fraction for NO_2_/O_3_ > 1, to qualitatively examine this effect. Figure S9 shows that the data fractions for NO_2_/O_3_ > 1 decreased from 32–39% in 2014 to 17–20% in 2022 in the three regions. In comparison, the corresponding fraction in CAREBEIJING-2007 data was 47%.

Figure S10 shows simulated hourly steady-state NO_3_ radial concentrations as a function of NO concentration using hourly averaged values of the O_*x*_, O_3_, and NO_2_ for the NE region from 2014 to 2002. The critical NO concentrations that suppress NO_3_ radicals were at the level of 0.1–1 ppbv. Similar results were obtained for the SE and NW regions. As NO_*x*_ emissions continuously decrease, it is plausible that in the future nighttime NO concentrations in certain urban regions may frequently decrease to levels below 0.1 ppbv. Under such circumstances, NO_3_ concentrations will increase by orders of magnitude (Figure S7).

In this study, we analyzed surface observations from 640 stations in China to characterize regional near-surface PNO_3_ changes from 2014 to 2022. It is worth noting that the differential optical absorption spectroscopy measurements by Yan et al. in Beijing showed increasing NO_3_ radical concentrations with altitude due to in part to increasing NO_3_ lifetime with altitude.^24^ Although not subject to regulatory monitoring, understanding the vertical distributions of O_3_, NO_2_, and other atmospheric species, along with their variations, is essential in gaining insights into nighttime oxidation processes.

### 3.4 Discussion

Satellite observations showed that NO_*x*_ emissions started decreasing by 2011 in southern China.^25^ The year 2013 marked a significant turning point in emissions reduction policies in China, leading to substantial decreases in NO_*x*_ emissions.^26^ Before 2013, the increased emissions led to O_*x*_ increases in the summer,^22,23^ likely leading to a PNO_3_ increase since it is a quadratic function of O_*x*_. The sensitivity of PNO_3_ to the NO_2_/O_3_ ratio is significantly lower than the quadratic dependence on the O_*x*_ (eq 1 and Figure 3). However, increased NO_*x*_ emissions also led to higher NO concentrations in urban regions near emission sources. The lifetime of the NO could be fairly long when the O_3_ was titrated through R1 by continuous emissions. Therefore, higher PNO_3_ concentrations during this period did not necessarily imply higher NO_3_ radical concentrations and nighttime oxidation in urban areas with high NO emissions (Figure S10).

After 2014, observations showed significant and consistent reductions of NO_2_ and PM2.5 in China (Figures 2 and S1). Nighttime O_*x*_ concentrations had transient increases peaking around 2017 and 2018 and then returned to and even went lower than the 2014 levels after 2020. The continuous decrease in the ratio of NO_2_/O_3_, on the other hand, contributed to a decrease in PNO_3_. This continuous decrease in PNO_3_ will be modulated by O_*x*_ variations due to meteorological conditions and O_3_ photochemistry.^9,22^ If the modulation effect by O_*x*_ in the future is neutral or negative as decreasing anthropogenic emissions cause O_*x*_ to stay near the same level or start decreasing, a PNO_3_ decreasing trend is expected.

The reduction in the NO_3_ radical concentration following the decrease in PNO_3_ is not always directly proportional. In regions with minimal impact from fresh NO emissions, the decrease in the level of NO_2_ resulting from emission reduction (Figure 2) generally led to a decline in the formation rate of N_2_O_5_. Additionally, the decrease in PM2.5 (Figure S1) and, consequently, the reduction in aerosol surface area tended to slow down the loss of N_2_O_5_ to HNO_3_ and ClNO_3_ on aerosols. Both factors would contribute to decreasing the loss of NO_3_ by heterogeneous N_2_O_5_ reactions on aerosols, resulting in an increase of NO_3_ loss by its oxidation of VOCs for a given PNO_3_ rate. The net effect would mitigate the potential decrease of the NO_3_ oxidation of VOCs due to decreased PNO_3_. The opposite mitigation effect would likely occur during the phase of increasing anthropogenic emissions.

In urban regions significantly affected by fresh NO emissions, on the other hand, much more drastic nighttime NO_3_ changes could be expected. If nighttime O_3_ titration by NO emissions and a long NO lifetime occurred over a sufficiently long time and large area of an urban region during the phase of increasing emissions, an “explosion” of nighttime NO_3_ radicals near the surface would likely occur during the period of emission reduction after 2013 when the NO lifetime shortened at night. As NO emissions continue to decrease, NO suppression of NO_3_ radicals would continue to weaken. Significant nighttime nitrate radical concentrations are necessary conditions for the formation of HNO_3_ from N_2_O_5_ hydrolysis^2^ and gaseous and particulate organic nitrates.^4^ Therefore, in an urban area with high NO_*x*_ emissions, an upsurge could exist from minimal nighttime inorganic and organic nitrate production to significant nighttime nitrate production as a result of efforts to reduce emissions. The analysis in this work targeted summer, but the probability for this mechanism to occur is higher in other seasons, particularly in winter, since the O_3_ concentrations tend to be highest in summer. This mechanism could help explain some of the observed lower nitrate reductions compared to other secondary aerosol components in Beijing from 2011/2012 to 2017/2018.^27^

## Supporting Information Available

Model computed nocturnal dry deposition velocity distributions for O_3_ and NO_2_ (Text S1); standard deviations of O_*x*_, O_3_, and NO_2_, yearly summertime averages of PNO_3_ and O_*x*_, model computed monthly mean dry deposition velocities for O_3_ and NO_2_, standard deviations of PNO_3_, summertime hourly average PM2.5 concentrations, summertime hourly average ERA5 temperature, summertime hourly average reaction rate constant of R1, hourly average concentrations of O_*x*_, O_3_, and NO_2_ during the CAREBEIJING-2007 campaign, data fractions of nighttime hourly NO_2_/O_3_ ratio >1 from 2014 to 2022, and model calculated hourly steady-state NO_3_ radical mixing ratio as a function of NO (Figures S1–S10). (PDF)
