# Supplementary material for: Decreasing Production and Potential Urban Explosion of Nighttime Nitrate Radicals amid Emission Reduction Efforts
Source: Environ Sci Technol. 2023 Dec 8;57(50):21306–12. doi: 10.1021/acs.est.3c09259 (PMC10734213; doi:10.1021/acs.est.3c09259)
Supplement: Supplementary file 1 — es3c09259_si_001.pdf [file es3c09259_si_001.pdf]

Decreasing production and potential urban explosion of nighttime nitrate radicals amid emission reduction efforts

Yuhang Wang[a,*], Shengjun Xi[a], Fanghe Zhao[a], Lewis Gregory Huey[a], Tong Zhu[b]

[a] School of Earth and Atmospheric Sciences, Georgia Institute of Technology, Atlanta, GA 30332, United States

[b] State Key Joint Laboratory of Environmental Simulation and Pollution Control, College of Environmental Sciences and Engineering, Peking University, Beijing 100871, China

*Corresponding author: Yuhang Wang (yuhang.wang@eas.gatech.edu)

Supporting Information

Supporting Text 1, Supporting Figures S1 to S10, and Supporting References

Supporting Text

Support Text 1. Model computed nocturnal dry deposition velocity distributions for O_3 and NO_2

The Regional chEmical trAnsport Model (REAM) was applied to compute dry deposition velocities for O_3 and NO_2 for July 2017. More detailed model information was described in previous publications (1-3). For dry deposition velocity calculation, the model is driven by assimilated meteorological fields from a weather research and forecasting (WRF) simulation constrained by the ERA5 reanalysis data (3). The resistance-in-series dry deposition module was the same as in the GEOS-Chem model (5).

The midnight average dry deposition velocity for O_x is ~0.2 cm s^{-1} for polluted eastern China (Figures S3 and 1). Given an O_x lifetime of ~20 hours (Figure 2), the estimate nocturnal boundary layer height is ~140 m if dry deposition is the only O_x sink. This estimated value is in qualitative agreement with measured values of 140-200 m in Beijing and Paris (6, 7). The midnight average dry deposition velocity for O_x is ~0.1 cm s^{-1} for the NW region, corresponding to an nocturnal boundary layer height estimate of ~100 m for an O_x lifetime of ~30 hours (Figure 2) if dry deposition is the only O_x sink. The lower estimated nocturnal boundary layer height in the NW than eastern China reflects in part considerably lower building height in the NW (8).

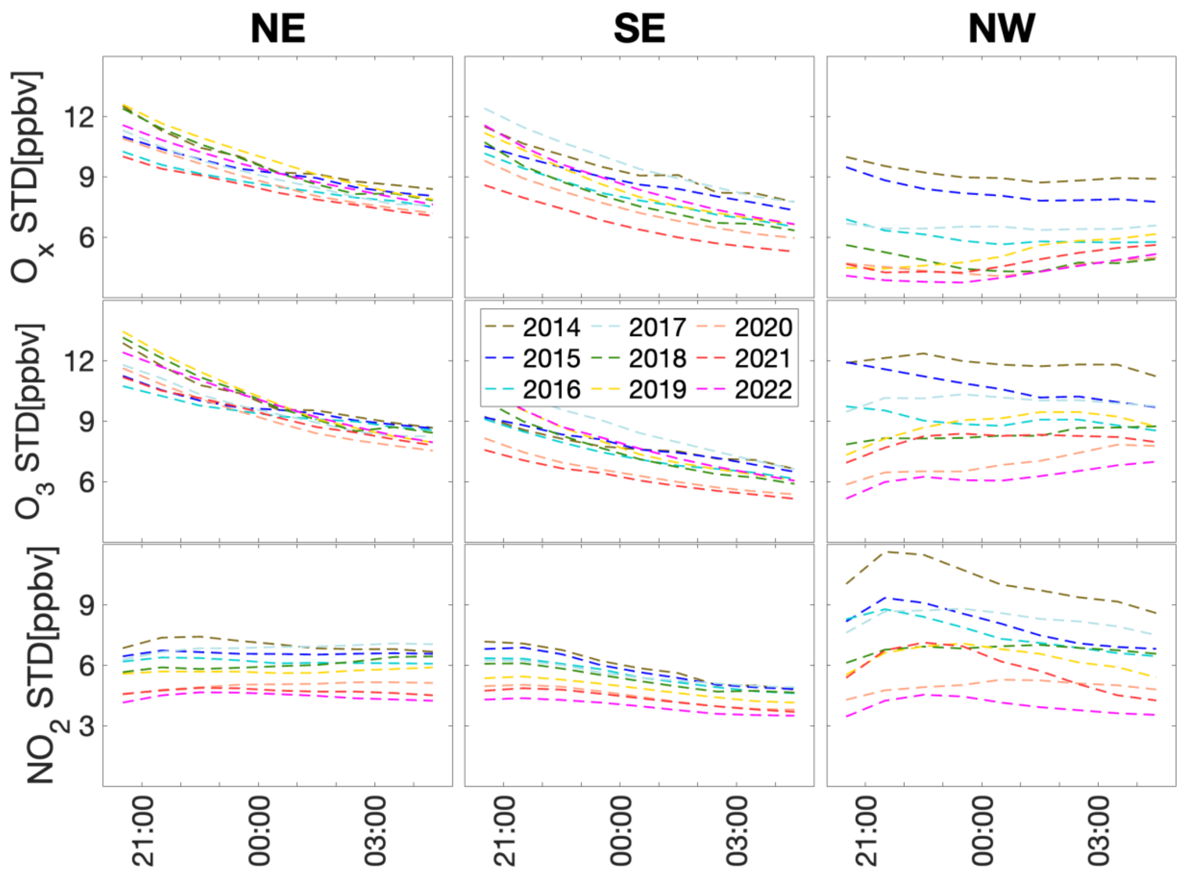

Figure S1. Same as Figure 1 but for the corresponding standard deviations among the observation sites.

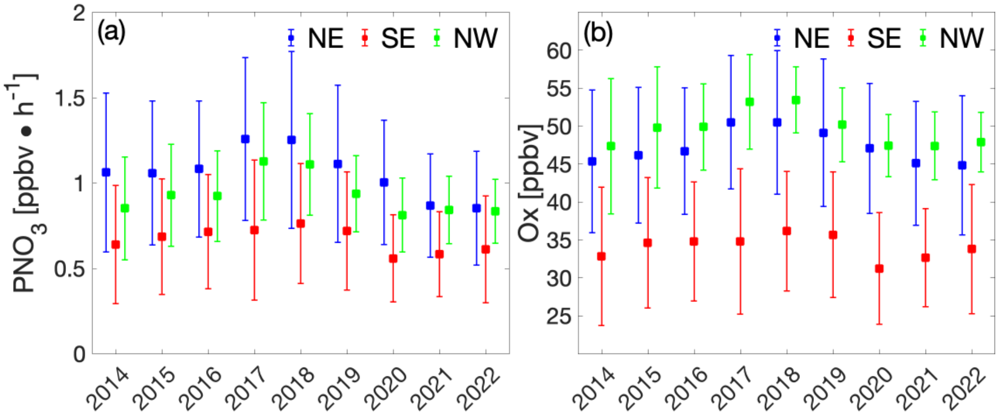

Figure S2. Yearly summertime averages of PNO_3 (a) and O_x (b) for the NE, SE, and NW regions from 2014 to 2022. The vertical bars show the standard deviations among the observation sites.

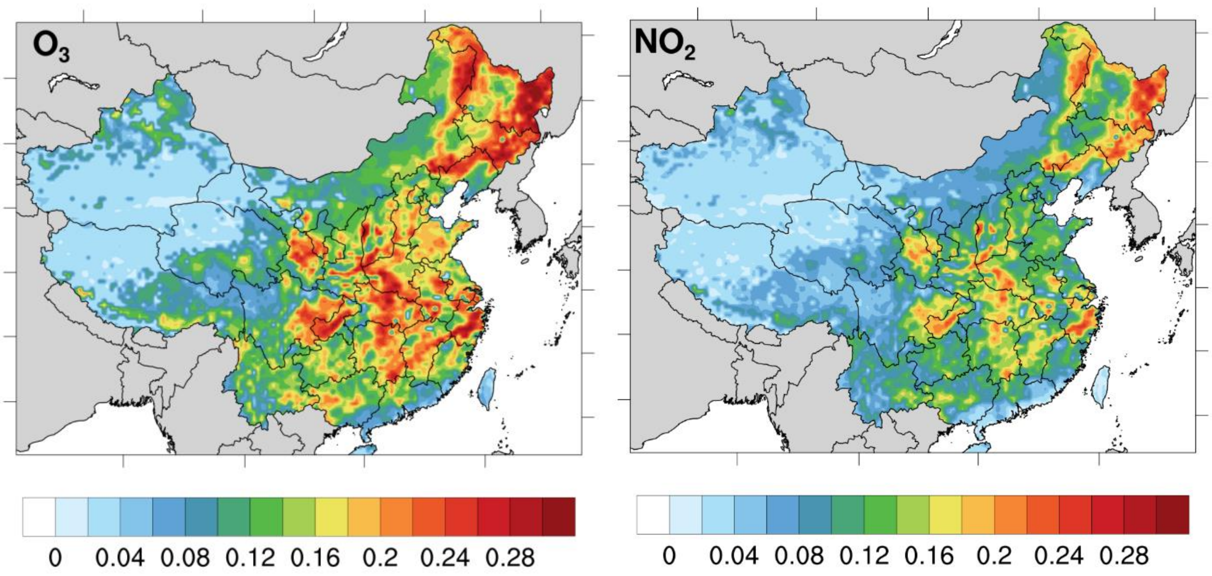

Figure S3. Model computed monthly mean dry deposition velocities (cm s^{-1}) for O_3 and NO_2 at mid night for July 2017.

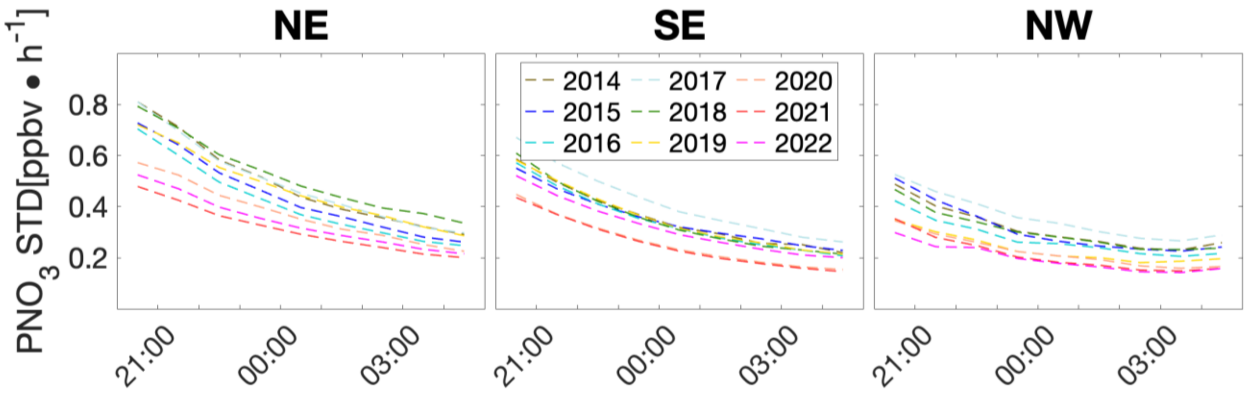

Figure S4. Same as Figure 4 but for the corresponding standard deviations of PNO_3 among the observation sites.

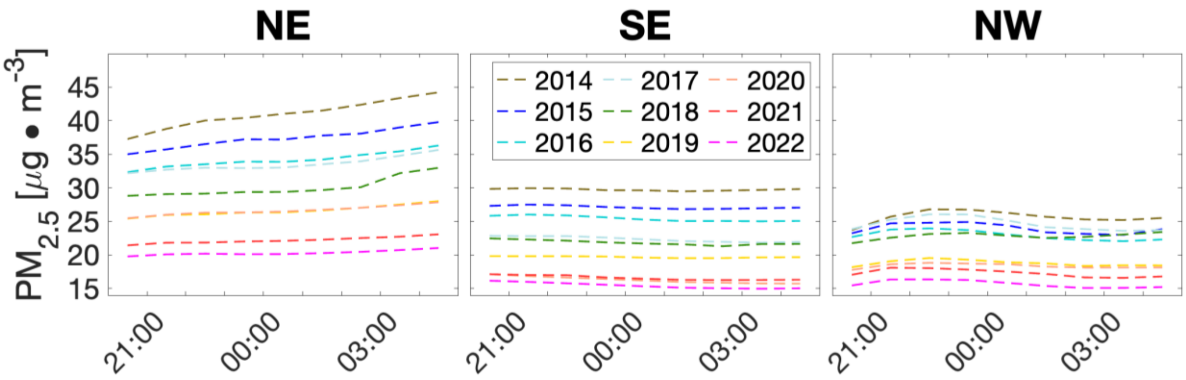

Figure S5. Same as Figure 2 but for summertime hourly average PM2.5 concentrations.

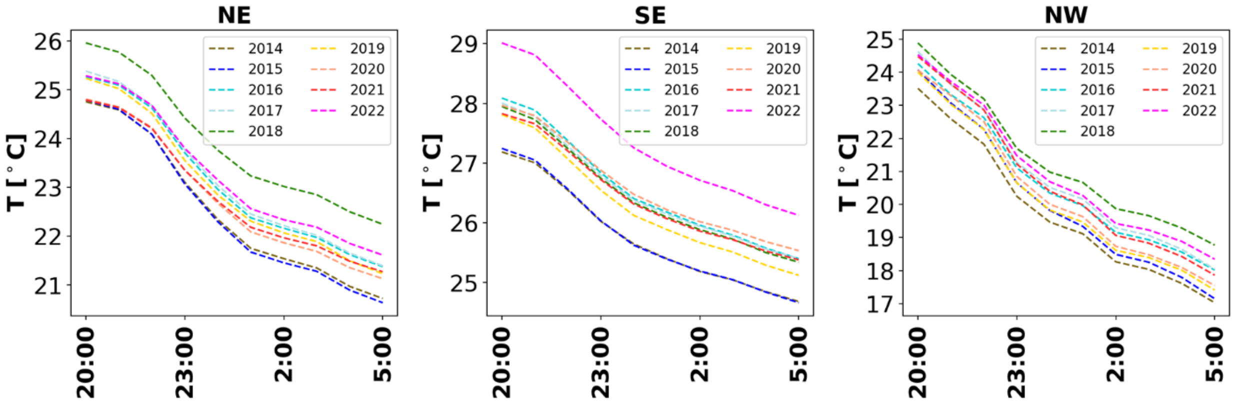

Figure S6. Same as Figure 2 but for summertime hourly average ERA5 temperature.

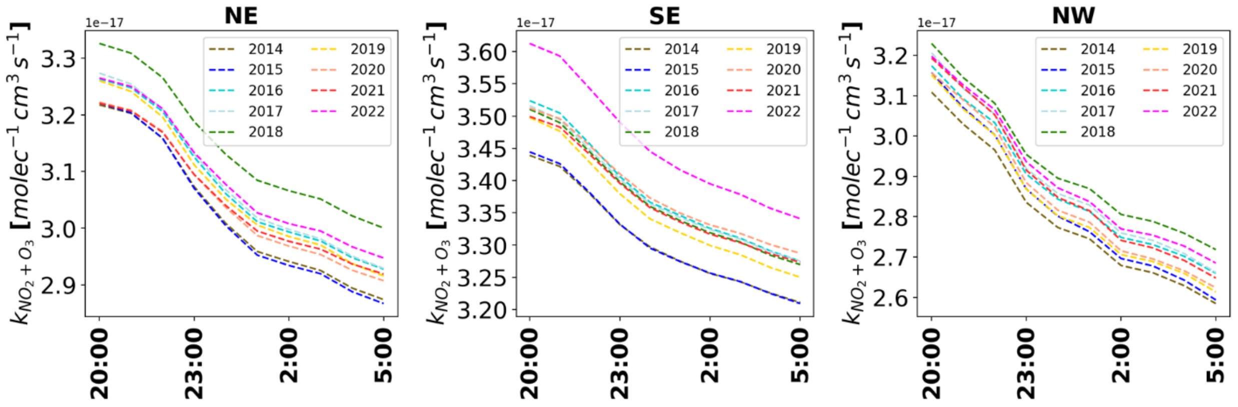

Figure S7. Same as Figure 2 but for summertime hourly average reaction rate constant of R1 using T data from Figure S6 (in the unit of 10^{-17} molec^{-1} cm^{3} s^{-1}).

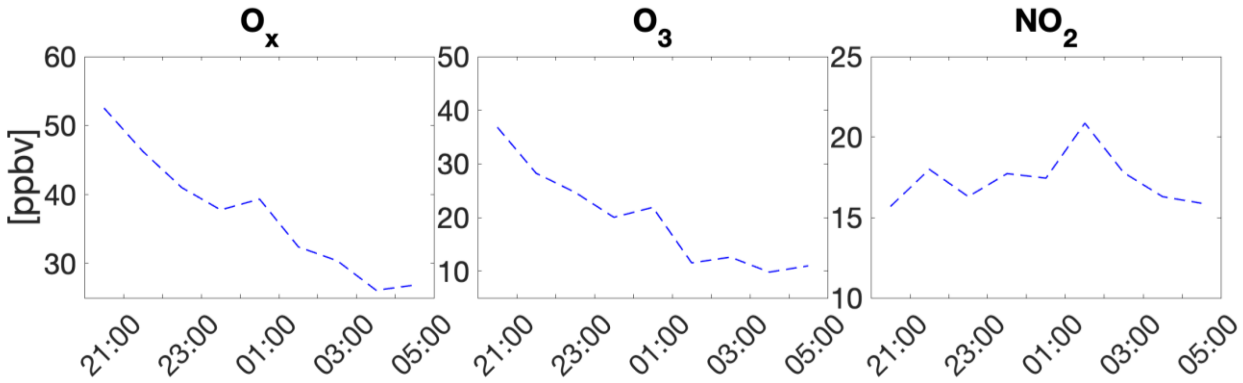

Figure S8. Same as Figure 2 but for hourly average concentrations of O_x, O_3, and NO_2 during the CAREBEIJING-2007 campaign.

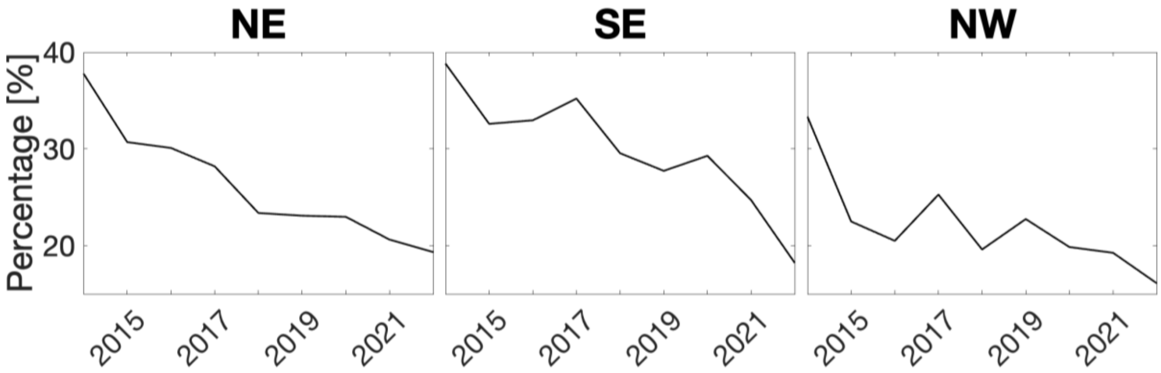

Figure S9. Data fractions of nighttime hourly NO_2/O_3 ratio > 1 from 2014 to 2022 in the NE, SE, and NW regions.

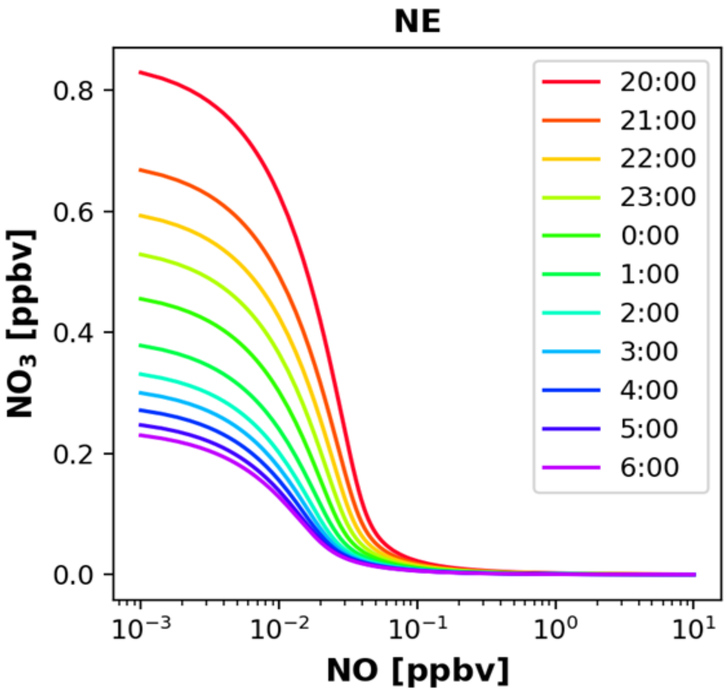

Figure S10. Model calculated hourly steady-state NO_3 radical mixing ratio as a function of NO for the NE region. Hourly average data for 2014-2022 are used in this calculation.

References

1. Qu, H., Wang, Y., Zhang, R., and Li, J., Extending ozone-precursor relationships in China from peak concentration to peak time (2020), J. Geophys. Res., 125, e2020JD033670, https://doi.org/10.1029/2020JD033670.
2. Li, J., Wang, Y., Zhang, R., Smeltzer, C., Weinheimer, A., Herman, J., Boersma, K. F., Celarier, E. A., Long, R. W., Szykman, J. J., Delgado, R., Thompson, A. M., Knepp, T. N., Lamsal, L. N., Janz, S. J., Kowalewski, M. G., Liu, X., and Nowlan, C. R. (2021), Comprehensive evaluations of diurnal NO_2 measurements during DISCOVER-AQ 2011: effects of resolution-dependent representation of NOx emissions, Atmos. Chem. Phys., 21, 11133–11160, https://doi.org/10.5194/acp-21-11133-2021, 2021.
3. Yan, Q., Wang, Y., Cheng, Y., Li, J. (2021), Summertime clean-background ozone concentrations derived from ozone precursor relationships are lower than previous estimates in the Southeast United States, Environ. Sci. Technol., 55, 12852−12861, doi:10.1021/acs.est.1c03035.
4. Hersbach, H., Bell, B., Berrisford, P., Hirahara, S., Horányi, A., Muñoz-Sabater, J., Nicolas, J., Peubey, C., Radu, R., Schepers, D., Simmons, A., Soci, C., Abdalla, S., Abellan, X., Balsamo, G., Bechtold, P., Biavati, G., Bidlot, J., Bonavita, M., De Chiara, G., Dahlgren, P., Dee, D., Diamantakis, M., Dragani, R., Flemming, J., Forbes, R., Fuentes, M., Geer, A., Haimberger, L., Healy, S., Hogan, R. J., Hólm, E., Janisková, M., Keeley, S., Laloyaux, P., Lopez, P., Lupu, C., Radnoti, G., de Rosnay, P., Rozum, I., Vamborg, F., Villaume, S., & Thépaut, J.-N (2020), The ERA5 global reanalysis, Quarterly Journal of the Royal Meteorological Society, 146, 1999–2049, https://doi.org/10.1002/qj.3803.

5. Bey, I.; Jacob, D. J.; Yantosca, R. M.; Logan, J. A.; Field, B. D.; Fiore, A. M.; Li, Q.; Liu, H. Y.; Mickley, L. J.; Schultz, M. G. (2001), Global modeling of tropospheric chemistry with assimilated meteorology: Model description and evaluation. J. Geophys. Res.: Atmos., 106, 23073−23095.
6. Yan, Y., Wang, S., Zhu, J., Guo, Y., Tang, G., Liu, B., An, X., Wang, Y., & Zhou, B. (2021). Vertically increased NO_3 radical in the nocturnal boundary layer. Science of The Total Environment, 763, 142969. https://doi.org/10.1016/j.scitotenv.2020.142969.
7. Pal, S., Xueref-Remy, I., Ammoura, L., Chazette, P., Gibert, F., Royer, P., Ravetta, F. (2012). Spatio-temporal variability of the atmospheric boundary layer depth over the Paris agglomeration: an assessment of the impact of the urban heat island intensity, Atmos. Environ. 63, 261–275, http://dx.doi.org/10.1016/j.atmosenv.2012.09.046.
8. Wu, W.-B., Man, J., Banzhaf, E., Meadows, M. E., Yu, Z.-W., Guo, F.-X., Sengupta, D., Cai, X.-X., Zhao, B. (2023). A first Chinese building height estimate at 10 m resolution (CNBH-10 m) using multi-source earth observations and machine learning, Remote Sensing of Environment, 291, 113578, https://doi.org/10.1016/j.rse.2023.113578.
